# Notes on Surgical Diseases of Children

**Published:** 1889-11

**Authors:** Edward Borck

**Affiliations:** St. Louis, Mo.


### Notes on Surgical Diseases of Children.

BY EDWARD BORCK, A M., M.D., ST. LOUIS, MO.

CONGENITAL.—*Hare-lip.*—For a single, simple hair-lip, after having tried the various methods known, I operate now with the object of not losing or destroying any tissue if possible. I insert a very fine needle-knife and pierce through the lower margin of one corner of the lip, sweeping the knife around to the opposite corner, thus leaving a complete and continuous bridge between the two parts of the lips. I then pull it down, uniting the wound with three or four cambric needles deeply inserted. Upon the needles a small piece of cork is put, on each side. A single figure-of-eight ligature is employed to hold it in its place, and the whole is penciled over with flexible collodion. In youths or adults I use small glass beads instead of cork, two or three on each needle. If swelling should occur I can break off one or more beads and thus relieve the tension. I prefer to operate as soon as possible; the earlier the better for the unfortunate creature, and the better success for the surgeon. The advantage of this plan is that the cork as well as the glass does not irritate the skin, and if any swelling should occur the ligature can be loosened without risk of separating the wound. The cork retracts slowly and enough to correct this swelling. Not dividing the lower margin in two nor sacrificing any tissue, leaves me a complete natural bridge, a great advantage in cases in which union by first intention is not obtained. It also fills up the gap better. The objections that one may raise who has never tried this plan may be that it leaves a pendulum or a flap hanging down; but if you have patience and wait, that which at first may seem a little unsightly to the eye, will in a few months disappear—so to say, it will be appropriated or absorbed by the surrounding tissue. If, however, there remains a small protrusion, it can easily be removed with the knife to suit the case, and a much better result is obtained.

In double, simple hare-lip, I operate on one side at a time. After a perfect or complete healing of the first, I operate on the second.

In simple, complete hare-lip, running into the nostrils, I operate in a similar way, paring the edges according to circumstances, let the flap hang down, and remove the superfluous portion, if any, some time after union has taken place.—*Medical and Surgical Reporter.*

